# Up-regulation of plasma membrane H^+^-ATPase under salt stress may enable *Aeluropus littoralis* to cope with stress

**Published:** 2014-03

**Authors:** Hosna Olfatmiri, Abbas Alemzadeh, Zahra Zakipour

**Affiliations:** Department of Crop Production and Plant Breeding, College of Agriculture, Shiraz University, Shiraz, Iran

**Keywords:** ABA, salinity, *GSTF1*, qRT-PCR, wheat

## Abstract

Most plants encounter stress such as drought and salinity that adversely affect growth, development and crop productivity. The expression of the gene glutathione-s-transferases (GST) extends throughout various protective mechanisms in plants and allows them to adapt to unfavorable environmental conditions. *GSTF1 *(the first phi GSTFs class) gene expression patterns in the wheat cultivars Mahuti and Alamut were studied under salt and ABA treatments using a qRT-PCR technique. Results showed that gene expression patterns were significantly different in these two cultivars. Data showed that in Mahuti, there was an increase of transcript accumulation under salt and ABA treatments at 3h, 10h and 72h respectively. In Alamut, however, the pattern of transcript accumulation was different; the maximum was at 3h. In contrast, there were no significant differences observed between the cultivars for *GSTF1* gene expression profiles at three levels of NaCl concentration (50, 100, and 200 mM) or in ABA (Abscisic Acid) treatment. It is likely that difference of gene expression patterns between the cultivars (Mahuti as a salt tolerant cultivar and Alamut as a salt sensitive cultivar) is due to distinct signaling pathways which activate *GSTF1* expression. Lack of a significant difference between the *GSTF1* gene expression profile under salt and ABA treatments suggests that the *GSTF1* gene is not induced by stress stimuli. Of course it is possible that other levels of NaCl and ABA treatments cause a change in the *GSTF1* gene.

## INTRODUCTION

World agriculture faces many problems and challenges, mostly caused by biotic and abiotic stresses such as salt stress. Concerns about the effect of salt stress on plants have come to dominate those regarding the environment [1, 2]. Much of the injury to plant cells caused by salt stress is associated with oxidative damage and ion homeostasis disruption during stress [3]. 

Plasma membrane (PM) H^+^- ATPases are major species of plasma membrane ion pumps [4], which are integral membrane proteins that use chemical energy to transfer protons to the extracellular environment by a primary active transport [4, 5]. PM proton pump is involved in many physiological processes in plants like salt tolerance [6, 7]. Recently, a number of studies have demonstrated that the expression of the PM H^+^-ATPase gene increased in response to salt stress [8, 9]. The way salt stress up-regulates the expression of PM H^+^- ATPase may be revealed by comparing halophytic and ordinary plants. 

Gouan, *Aeluropus littoralis*, is a monocotyledonous angiosperm that thrives in salt areas [10]. Several studies have been carried out on salt-resistance in gouan but little is known about the mechanism. In other halophytes, the activity of PM H^+^-ATPase has been shown to increase under salt stress [11]. It has also been shown that PM H^+^-ATPase activity in *Zostera marina*, a marine halophytic plant, was not inhibited by NaCl treatment, but PM ATPase activities were inhibited in rice or freshwater grass [12]. Mature leaves of halophytes have also been reported to have protoplasts with high ATPase activity, which increases the capacity of ion flux between the inside and outside of the cell [13, 14]. These results indicate that salt tolerant ATPase activity in the plasma membrane must play a critical role in the regulation of ion homeostasis under salt stress in halophytic plants.

The aim of the present work was to examine the effect of salt on the expression pattern of PM H^+^-ATPase gene in halophyte *A. littoralis*. The correlation between morphological changes in leaves and the expression pattern of PM proton pump gene were also studied.

## MATERIALS AND METHODS

**Plant materials:** *A. littoralis* seeds were obtained from Pakan Seed Research Center, Isfahan, Iran. The seeds were surface sterilized by soaking in 1% (v/v) sodium hypochlorite for 20 min and rinsed several times with distilled water. They were then put in Petri dishes containing 2 moist filter paper sheets and germinated in an incubator at 25 ºC. The germinated seeds were transplanted into pre-compressed Jiffy pots soaked in water, and incubated in a growth chamber (with 25:16 ºC day:night temperature, 16 h light/8 h dark photoperiod) and irrigated daily with ½ MS nutrient solution [15].

**NaCl treatment: **After two months, plants were treated with NaCl at different concentrations for 48 h. To prevent osmotic shocks, NaCl was added gradually (50 mM every day) to plants up to the final concentrations of 0, 50, 100, 150, 200, 250, 500 or 1000 mM. The experiment was carried out in a completely randomized design with 3 replicates. 

**Primer design: **Gene coding sequences for PM H^+^-ATPase (accesstion number: AB686268) and actin (accesstion number: FJ603097) were obtained from the NCBI website. Two-pair primers were designed based on the sequences of these genes using Vector NTI (Version 9) program (Table 1). 

**Table 1:** The sequences of primers used to amplify the genes encoding a plasma membrane H^+^-ATPase (target gene) and actin as reference gene in Q-PCR

**Gene**	**Name of** **Primer**	**Sequence**	**TM** **(°C)**
target gene	PMH1RTPF	5'- ACCTGAGAAGACCAAGGAGTCT-3'	62.15
	PMH1RTPR	5'- TACAGGAAGTGCTTCAAGTGTAG-3'	60
actin gene	ActinAlF	5'- CGTACAACTCCATCATGAAGTG-3'	61.96
	ActinAlR	5'- CAAACACTGTACTTTCTCTCCG-3'	60.35

**RNA extraction and cDNAs synthesis: **Total RNA was isolated from *A. littoralis* leaves using RNeasy Plant Mini Kit (Qiagen, USA) according to the manufacturer’s instructions. The quality of RNA was assessed by electrophoresis on agarose gel. cDNAs were reverse transcribed from the total RNA extracted from leaves using SuperScript II Kit (Invitrogen, USA) with oligo(dt) primer [16]. 

**RT-PCR: **cDNAs were amplified by PCR using a specific primer for the gene encoding plasma membrane proton pump. PCR reactions were carried out in a final volume of 20 µl reaction mixture containing 10 mM Tris (pH 8.3), 1.5 mM MgCl_2_, 50 mM KCl, 200 µl dNTPs, 0.3 µM of each primer and 1 unit of ExTaq DNA polymerase (Takar, Japan) under following conditions: 5 min 94ºC followed by 30 cycles at 94ºC for 30s, 60ºC for 1 min, and 72ºC for 5 min, with a final extension at 72ºC for 15 min. 

**Quantitative real-time PCR: **PCR reactions were carried out in a final volume of 10 µl containing 5 µl of SYBR Premix ExTaq (Takar, Japan), 1 µl cDNAs and 0.3 µM of each primer (Table 1) under the following conditions: 1 min at 94ºC, 45 cycles at 94ºC for 15s, 60ºC for 15s, and 72ºC for 30s. At the end of the program, the specificity of the primers’ set was confirmed by melting curve analysis (65-95ºC with a heating rate of 0.5ºC/min). The copy numbers of the genes’ mRNAs were estimated by comparing the results of real-time PCR with serial dilutions (10^1^, 10^2^, 10^3^, 10^4^, 10^5^, and 10^6^ copies/µl) of the plasmid containing amplified fragments of each gene. The gene which encodes actin was used to normalize the expression ratio of the target gene.

## RESULTS AND DISCUSSION

**Figure 1:**
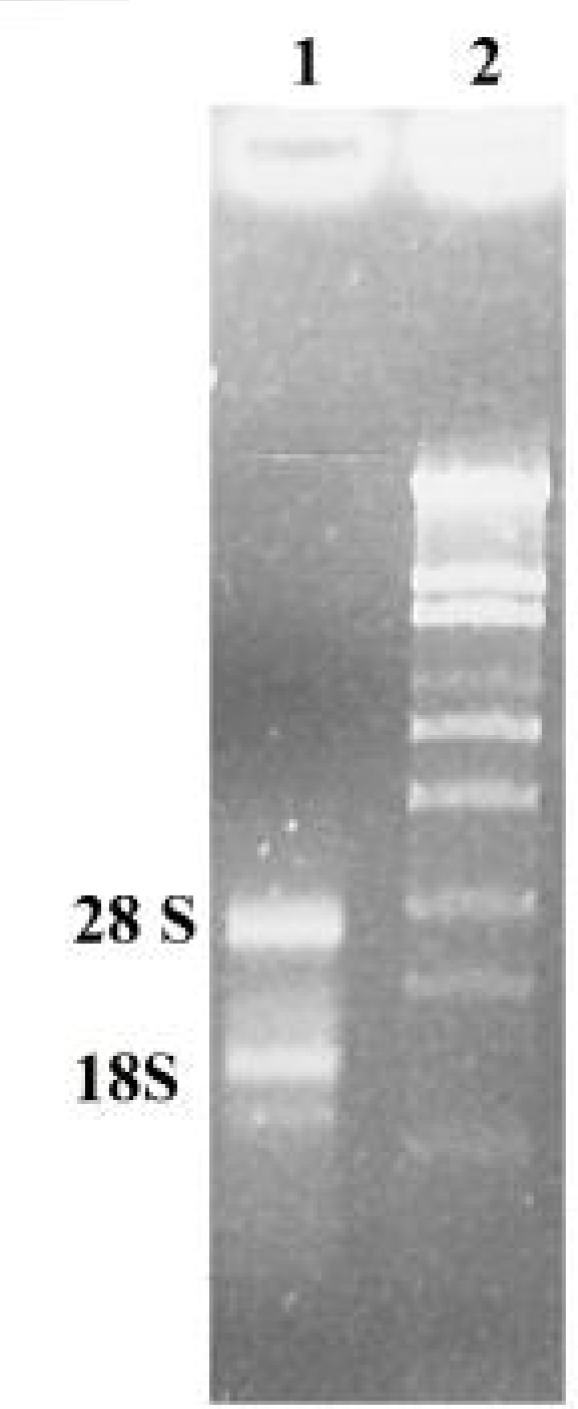
Total RNA extracted from leaves of *A. littoralis* was separated by 1% agarose gel electrophoresis and stained with ethidium bromide. 1) Total RNA extracted from sample and 2) size marker λ/*Sty*I.

The quality of the extracted RNA was determined by electrophoresis on 1% agarose gel. The OD260/OD280 ratio of the extracted RNA was 1.8. Bands corresponding to 18S and 28S rRNA were distinctly visible on the gel, indicating high quality, non-degraded RNA (Fig. 1).

**Figure 2:**
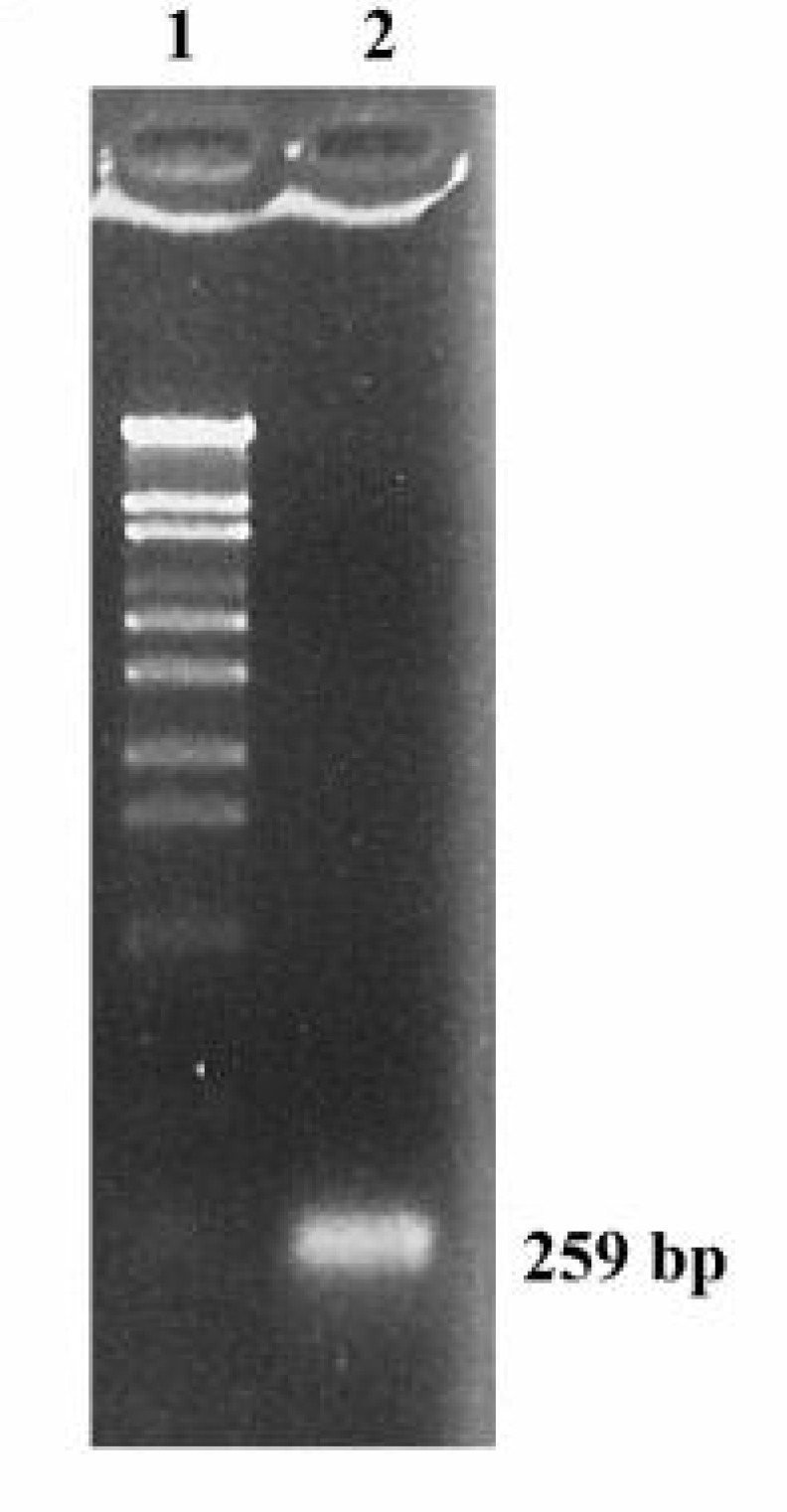
Agarose gel electrophoretic analysis of RT-PCR-amplified cDNA of a plasma membrane proton pump gene. 1) Size marker λ/*Sty*I and 2) A 259 bp fragment was amplified from cDNAs prepared from RNA of leaves

Since RT-PCR is a reaction sensitive to the presence of inhibitors or the degradation of RNA, cDNAs prepared from RNA were used as template in a standard PCR reaction to test RNA quality. A 259 bp fragment was amplified with a specific primer for the PM proton pump gene (Fig. 2) which indicated the high quality of the prepared cDNAs. 

**Figure 3:**
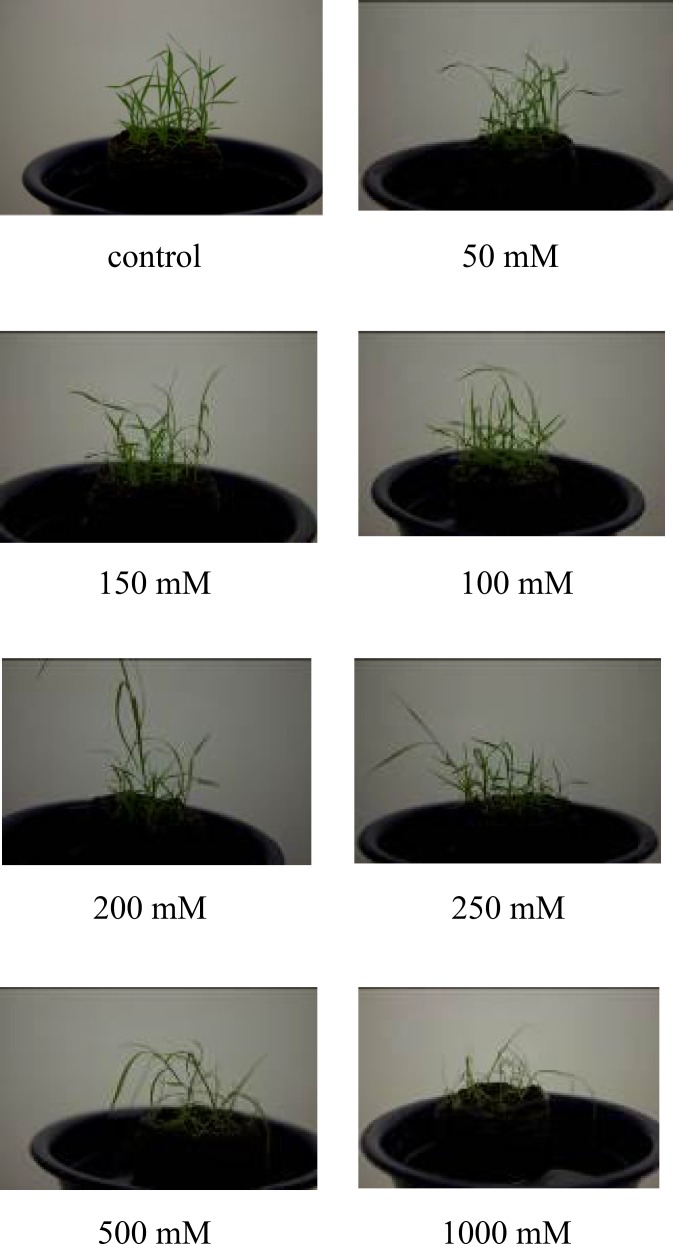
*A. littoralis* plants were treated by different concentrations of NaCl (control, 50, 100, 150, 200, 250, 500, 1nd 1000 mM

**Figure 4:**
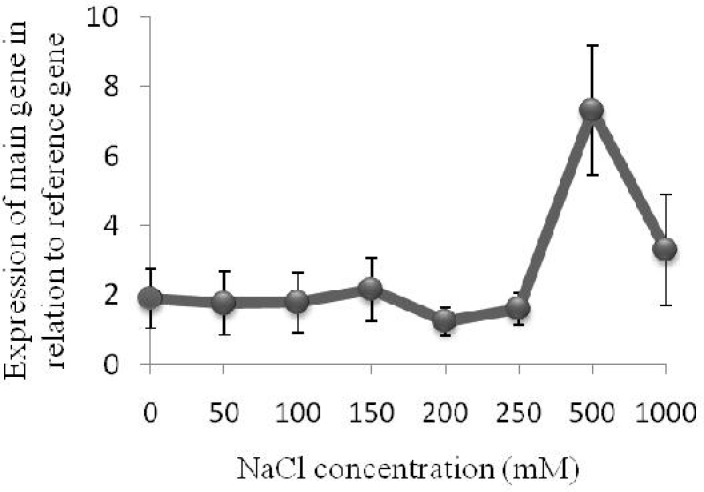
Quantitative Real-time PCR analysis. The relative expression level of a gene encoding plasma membrane proton pump in leaves of *A. littoralis* (normalized by the level of actin gene, as a housekeeping gene) is shown

The results also demonstrate a lack of significant changes in the expression level of the gene encoding PM H^+^-ATPase in *A. littoralis* with increasing NaCl concentrations; however, this expression suddenly increased at 500 mM, followed by a dramatic decrease at 1000 mM (Fig. 4). It may be suggested that the cells of this plant, which must be salt tolerant through intrinsically cellular mechanisms, regulate the expression of the proton pump gene in response to NaCl to accommodate the solute accumulation necessary for osmatic adjustment. Since the Na^+^ ion is one of the solutes toxic to cytosolic metabolisms, it must be extruded out of the cell by a PM Na^+^/H^+^ antiporter.

As mentioned above, morphological changes were also induced at 500 mM, suggesting that proton pump in *A. littoralis *has an important role in its response to salt stress and that a reduction of its expression can result in a wide variety of morphological changes. It has been previously shown that the expression of genes which encode H^+^-ATPase pumps in different plants increased in response to salt stress [8, 21, 22]. Similarly, our results showed the expression pattern of H^+^-ATpase gene to be affected by salt stress; yet, no other report has indicated the expression of the PM proton pump gene to increase markedly in high concentrations of NaCl (500 mM) or to decline under still higher concentrations (1000 mM). Although the expression of some genes such as *Tsvp*, a gene for H^+^-pyrophosphatase in *Thellungiella halophila*, was shown to have increased during the first 16 h under high concentrations of NaCl (300 mM) and decreased after 16 h, the way the expression was regulated under high concentrations of NaCl was not investigated [23]. 

It can be suggested that *A. littoralis* cells regulate the expression of the plasma membrane proton pump gene in response to NaCl to generate a proton motive force (PMF). PMF is the source of energy for a variety of cellular proteins such as Na^+^/H^+^ antiporters [24]. Despite the fact that toxic ions such as Na^+^ would be compartmentalized in the vacuole by tonoplast antiporters and pumps [25], extruding them out of the cell across the plasma membrane is critical to the maintenance of ionic homeostasis in the cytosol carried out by plasma membrane antiporters and pumps [24, 26]. The activity of PM Na^+^/H^+^ antiporters in *A. littoralis* cells lacking Na^+^ ATPase activities induces the ability of these cells to export Na^+^ ions and greatly increase their resistance to salinity [27]. It has been shown that small changes in proton pump activities are important in plant growth and abiotic stress tolerance [28]. It can be thus suggested that salt stress triggers the expression of the PM proton pump to provide the driving force for Na^+^ exclusion. In other words, PM H^+^-ATPase and Na^+^/H^+^ antiporters may coordinate the regulation of salt tolerance. 

It seems that the activity of this protein is essential for ion homeostasis in *A. littoralis* under 500 mM NaCl, but under a higher 1000 mM concentration when the plant needs more energy, the quantity of this protein decreases to minimize energy expenditure by proton pumps, thus increasing fitness. In addition, it is possible for cell membranes to be destroyed by exposure to high concentrations of NaCl, decreasing the necessity of proton pump and its expression. It is shown in figure 3 that plants were severely affected by salt stress at 1000 mM NaCl.

Enhanced expression of the PM proton pump gene was detected in *A. littoralis* leaves in 500 mM of NaCl, but not in the higher concentration (1000 mM). The high degree of NaCl induction of PM H^+^-ATPase mRNA accumulation in *A.* *littoralis* leaves indicates a requirement for this pump in the cells during salt adaptation. Our data revealed that the halophyte *A. littoralis’ *was fast to respond to NaCl by inducing the PM proton pump gene expression. This result is consistent with a previous report that showed a rapid turnover of the PM proton pump in maize [29]. 

On the basis of our results, a correlation between salt-induced morphological changes and the expression level of the PM proton pump gene in *A. littoralis* may be also suggested. The great capacity of halophyte *A. littoralis *to induce the PM proton pump gene in response to high concentrations of NaCl indicates the presence of aunique regulatory elements and/or transcription factors that are highly responsive to salt stress.
